# Praziquantel-encapsulated niosomes against *Schistosoma mansoni* with reduced sensitivity to praziquantel

**DOI:** 10.7705/biomedica.5913

**Published:** 2022-03-01

**Authors:** Eglal I. Amer, Iman F. Abou-El-Naga, Laila M. Boulos, Heba S. Ramadan, Salwa S. Younis

**Affiliations:** 1 Medical Parasitology Department, Faculty of Medicine, Alexandria University, Alexandria, Egypt Alexandria University Medical Parasitology Department Faculty of Medicine Alexandria University Alexandria Egypt; 2 Medical Bio-Physics, Medical Research Institute, Alexandria University, Alexandria, Egypt Alexandria University Medical Bio-Physics, Medical Research Institute Alexandria University Alexandria Egypt

**Keywords:** Schistosoma mansoni, drug resistance, liposomes, praziquantel, Schistosoma mansoni, resistencia a medicamentos, liposomas, praziquantel

## Abstract

**Introduction:**

Praziquantel (PZQ) is the only commercially available drug for schistosomiasis. The current shortage of alternative effective drugs and the lack of successful preventive measures enhance its value. The increase in the prevalence of PZQ resistance under sustained drug pressure is, therefore, an upcoming issue.

**Objective:**

To overcome the tolerance to PZQ using nanotechnology after laboratory induction of a *Schistosoma mansoni* isolate with reduced sensitivity to the drug during the intramolluscan phase.

**Materials and methods:**

Shedding snails were treated with PZQ doses of 200 mg/kg twice/ week followed by an interval of one week and then repeated twice in the same manner. The success of inducing reduced sensitivity was confirmed *in vitro* via the reduction of cercarial response to PZQ regarding their swimming activity and death percentage at different examination times.

**Results:**

Oral treatment with a single PZQ dose of 500 mg/kg in mice infected with cercariae with reduced sensitivity to PZQ revealed a non-significant reduction (35.1%) of total worm burden compared to non-treated control mice. Orally inoculated PZQ- encapsulated niosomes against *S. mansoni* with reduced sensitivity to PZQ successfully regained the pathogen’s sensitivity to PZQ as evidenced by measuring different parameters in comparison to the non-treated infected animals with parasites with reduced sensitivity to PZQ. The mean total worm load was 1.33 ± 0.52 with a statistically significant reduction of 94.09% and complete eradication of male worms. We obtained a remarkable increase in the percentage reduction of tissue egg counts in the liver and intestine (97.68% and 98.56%, respectively) associated with a massive increase in dead eggs and the complete absence of immature stages.

**Conclusion:**

PZQ-encapsulated niosomes restored the drug sensitivity against laboratory- induced *S. mansoni* adult worms with reduced sensitivity to PZQ.

## Resumen

**Introducción.:**

El prazicuantel es el único fármaco disponible comercialmente para la esquistosomiasis. La escasez actual de medicamentos alternativos y la falta de medidas preventivas eficaces aumentan su valor. La creciente prevalencia de la resistencia al prazicuantel bajo una presión prolongada del fármaco es, por tanto, un tema emergente.

**Objetivos.:**

Superar la tolerancia al prazicuantel mediante nanotecnología después de la inducción en laboratorio de un aislamiento de *Schistosoma mansoni* con sensibilidad reducida al fármaco durante la fase intramolusco.

**Materiales y métodos.:**

Los caracoles que liberaban cercarias se trataron con prazicuantel en dosis de 200 mg/kg dos veces por semana, seguidas de un intervalo de una semana, y luego se repitieron dos veces de la misma manera. La inducción exitosa de la sensibilidad reducida se confirmó *in vitro* mediante la reducción de la reacción de las cercarias al prazicuantel con respecto a su actividad de natación y el porcentaje de muerte en diferentes momentos de examen. El éxito en inducir una menor sensibilidad se confirmó *in vitro* mediante la reducción de la reacción de las cercarias al prazicuantel.

**Resultados.:**

El tratamiento oral con una dosis única de prazicuantel de 500 mg/kg en ratones infectados con cercarias con sensibilidad reducida al prazicuantel, reveló una reducción no significativa (35,1 %) de la carga total de gusanos en comparación con los ratones de control no tratados. Los niosomas encapsulados en prazicuantel inoculados por vía oral contra *S. mansoni* con sensibilidad reducida al prazicuantel, permitieron reestablecer con éxito la sensibilidad del patógeno al medicamento, como lo demostró la medición de diferentes parámetros en comparación con los animales infectados no tratados con parásitos con sensibilidad reducida a prazicuantel. La carga media total de gusanos fue de 1,33 ± 0,52, con una reducción estadísticamente significativa del 94,09 %, y la erradicación completa de los gusanos machos adultos. Se obtuvo un aumento notable en el porcentaje de reducción del recuento de huevos en el tejido del hígado y el intestino (97,68 % y 98,56 %, respectivamente), asociado con un aumento masivo de huevos muertos y ausencia total de estadios inmaduros.

**Conclusión.:**

Los niosomas encapsulados en prazicuantel restauraron la sensibilidad al fármaco contra gusanos adultos de *S. mansoni* con sensibilidad reducida al prazicuantel inducida en el laboratorio.

Schistosomiasis is a major health problem in tropical and sub-tropical areas. The global health burden of schistosomiasis is estimated at 3.3 million disability-adjusted life years (DALY), a value similar to that of malaria and tuberculosis ^1^. Chemotherapy remains the primary intervention for the disease ^2^ and since praziquantel (PZQ) is essentially the only drug currently available ^3^, such reliance on a single drug for a disease of this magnitude is undesirable, particularly in light of reports concerning schistosome isolates having reduced susceptibility to PZQ in the field ^4^^,^^5^.

Drug resistance depends on the selective pressure of drug exposure ^6^. Unfortunately, the mechanism of action of PZQ is not yet completely understood ^7^. Therefore, the PZQ tolerance mechanism and the methods for overcoming it remain unclear. To surpass this knowledge gap, laboratory- induced schistosome isolates with reduced sensitivity to PZQ permit the comparison between them and susceptible parasites. Under laboratory conditions, induction of resistance has been achieved via two approaches in either the definitive or intermediate hosts. In the first approach, mice infected with *S. mansoni* were initially treated with sub-curative doses of PZQ followed by increasing the dose in animals for several passages in mice/snails to complete the parasite’s life cycle ^8^^,^^9^. The second approach involves selecting PZQ-resistant parasites during the asexual stages of the life cycle of the snail intermediate host ^10^^,^^11^. An important mechanism of PZQ resistance involves an increase in its efflux by the multidrug resistance (MDR) transporters, of which glycoprotein transporters like SmMDR2 and multidrug resistance-like proteins (SmMRP1) are deemed important ^12^.

Many strategies including nanotechnology have been adopted to increase drug effectiveness ^13^. Lipid-based nanoparticles including liposomes, solid lipid nanoparticles, and nanostructured lipid carriers (the second generation of solid lipid nanoparticles) are promising oral drug-delivery candidates for PZQ ^14^^-^^16^. Furthermore, to avoid MDR, nanotechnology provides an innovative and promising alternative to conventional chemotherapeutics. Successful examples of nanotechnology use in reversing drug resistance and regaining drug activity include studies conducted with antibiotics ^17^^,^^18^, anticancer ^19^, and antimalarial drugs ^20^. Recently, the co-delivery of chemotherapeutics and inhibitors of multidrug resistance transporters using lipid-based nanocarriers provided a promising approach for overcoming drug resistance and reducing the potential toxicity of chemotherapeutic drugs in different diseases ^21^. Niosomes are among the best lipid carriers. They are widely used as alternatives to liposomes and are even preferred over them due to their high chemical stability and low cost. Moreover, niosomes possess other characteristics that make them promising candidates for clinical use ^22^ as they improve the oral bioavailability of drugs, enhance their dissolution rate, and protect them from being prematurely degraded/inactivated in addition to their known low toxicity and non-immunogenicity ^23^. Niosomes are essentially composed of cholesterol and non-ionic surfactant vesicles which trap and retain the aqueous solution of the solute particles. Besides, various ionic amphiphiles are incorporated into their structure to achieve stability by inducing negative or positive charges ^24^. This unique structure allows for the incorporation of hydrophilic drugs into its aqueous core and lipophilic drugs into its membrane bilayer ^25^. Thus, we selected them as a carrier system for overcoming reduced sensitivity to PZQ primarily due to their unique character of surfactant inclusion which sensitizes resistant cells and inhibits glycoprotein efflux transporters ^26^ and may help in expanding the spectrum of niosome-loaded drugs for treating resistant organisms in the future. Here, we induced in the laboratory reduced sensitivity to PZQ in the *S. mansoni* asexual stages inside a *Biomphalaria alexandrina* snail host and we evaluated the effect of PZQ-loaded niosomes in mice infected with an *S. mansoni* isolate with reduced sensitivity to PZQ taking advantage of niosomes potential as drug susceptibility enhancers*.*

## Materials and methods

**Figure 1:**
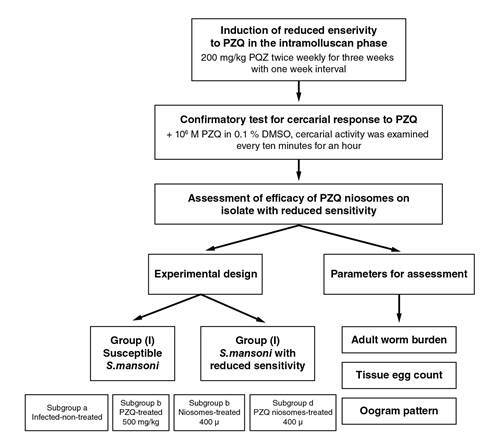
Flowchart showing a brief summary of the methodology carried on in this work.

We present a brief summary of the methodology used in the study in figure 1.

### Animals and parasites

We used an Egyptian strain of *S. mansoni* in all the experiments and ten Swiss-strain albino mice aged 4-6 weeks weighing 20-30 g and previously infected with *S. mansoni* from the Schistosome Biologic Supply Programme (SBSP) at the Theodor Bilharz Institute (TBI), Giza, Egypt.

The use of laboratory animals complied with the Egyptian national animal welfare standards and was approved by the Faculty of Medicine Ethics Committee at Alexandria University, Egypt (protocol approval number: 020732).

Snails and inbred Swiss albino mice, as well as the *S. mansoni* life cycle, were maintained at the Animal Unit of the Department of Medical Parasitology at Alexandria University. Seven weeks post-infection (p.i.), the mice were administered intraperitoneal cal-heparin (5,000 IU/ ml; 0.1 ml each) and euthanized by ether; their livers were used as a source of parasite eggs for snail infection ^27^.

### Snail source, maintenance, and infection

Four hundred laboratory-bred susceptible *B. alexandrina* snails were supplied by SBSP/TBRI and used for inducing PZQ reduced sensitivity during the intra-molluscan phase and harvesting cercariae for the *in vitro* animal infection and study. Snails were maintained in transparent plastic aquaria each containing 50 snails at 26-28°C in an incubator. Each container was filled with five liters of well-aerated aged dechlorinated tap water (DTW) and replaced twice a week. Freshly washed lettuce leaves were supplied as snail food every couple of days and soft chalk was added to all aquaria. Dead snails were regularly removed. Pieces of foam were placed inside the containers for egg deposition ^28^.

Seven weeks post-infection, mice were sacrificed and their livers used as a source of parasite eggs for snail infection. The collected eggs were exposed to light to stimulate miracidia release. The snails were individually exposed to 8-10 vigorously swimming, freshly-hatched miracidia in direct sunlight for 3-4 hours, and then the snails were kept in the dark and maintained under the conditions previously described ^29^.

### Induction of reduced sensitivity to PZQ in the intra-molluscan phase

*Preparation and administration of the drug.* To induce PZQ reduced sensitivity, we used 100 g of 99.5% pure PZQ white powder (C19H24N2O2) with a molecular weight of 312.40606 g/mol (Alexandria Company for Pharmaceuticals & Chemical Industries, Egypt). The drug was incorporated into mouse chow (purchased from the local market) ^11^ ground up with calcium carbonate in a 9:1 ratio. The ratio was reconstituted with water until it became pasty. The snails were individually weighed to calculate the drug dosage for each of them. PZQ was administered in doses of 200 mg/kg twice/ week followed by an interval of one week and then repeated twice in this same manner ^30^. The PZQ dose per kg was incorporated into 100 mg of food (the amount administered to each snail). Snails were allowed to consume completely the daily amount of food suggesting they received the entirety of the drugs offered in the ration ^10^.

The snails infected with *S. mansoni* were checked for cercarial shedding four weeks after infection. Two hundred shedding snails were divided equally into two groups. Group A contained the infected PZQ-unexposed snails used as a source of PZQ-susceptible cercariae while group B contained the infected PZQ-exposed snails. Both snail groups were maintained in glass containers and DTW was changed every 24 hours.

### Cercaria harvest

Cercaria from the *B. alexandrina* snails infected with the Egyptian *S. mansoni* strain in each group were harvested to check the response to PZQ *in vitro* and evaluate its efficacy against adult *S. mansoni* with reduced sensitivity to its action in animal models using nanotechnology. Each snail was placed in a 200 ml beaker containing dechlorinated tap water (DTW) at 30°C and kept under intense illumination at a distance of 50 cm from the light source. After two hours, the cercarial suspension was collected and 50 µl were stained with Lugol iodine to facilitate the count under a stereo microscope while the remaining cercarial suspension was used to complete the study ^31^.

### Confirmatory test for cercarial response to PZQ

A stock solution of 0.1% dimethyl sulfoxide (DMSO) in distilled water was used as a drug solvent and a control. Solutions of 5 x 10-6 M PZQ in 0.1 % DMSO were prepared and stored for a maximum of two weeks in the refrigerator. The assay was performed using ordinary glass slides without a coverslip. Three sets of slides were used for each group. For each set, the number of cercariae was 5-7 in 20 μl of DTW per drop and we used two drops per slide (36 *S. mansoni* cercariae). The first set of cercariae received no drug, the second received 20 μl of 0.1% DMSO per drop, and the third 20 μl of the PZQ solution per drop. Cercariae were examined under an ordinary microscope every ten minutes for one hour and carefully classified as unaffected, affected, or dead.

To avoid the slides drying up and the resulting increase in the PZQ concentration, and to stabilize the temperature during the 60-minute examination period, the slides were kept in a plastic box on a thin (3 mm) wet sponge. The temperature of the sponge was adjusted to 28oC by adding warm water ^32^. This procedure was performed in triplicate.

### Efficacy of PZQ on adult S. mansoni with reduced sensitivity to PZQ using nanotechnology

*Drug preparation.* For the PZQ suspension, 500 mg of PZQ powder were dissolved in one ml 60% ethanol and then suspended in a phosphate buffer saline (7 ml) to obtain a total volume of 8 ml. Each mouse was administered a single dose of PZQ (500 mg/kg) as 0.2 ml PZQ suspension ^33^.

### Preparation and characterization of PZQ-encapsulated niosomes

Niosomes were prepared via the thin-film hydration method; Span 60 and cholesterol in a 7:6 molar ratio were dissolved in 10 ml of chloroform and ethanol mixture (7/3, v/v) as an organic solvent in a round-bottom flask of a rotator evaporator. The solvent from the nanodroplets was extracted via evaporation at 55°C in the rotator evaporator under reduced pressure (200 mmHg) for 15 minutes until a thin film appeared in the inner wall of the flask leading to the formation of nanoparticles by precipitating macromolecules. The dry lipid film was hydrated with 10 ml PBS (pH 7.4) shaken for 15 minutes at low speed and at 55°C and then hand-shaken for 15 minutes at room temperature to obtain a lipid suspension**.** The particles were then downsized by sonication via a bath-type sonicator operated at a frequency of 55 kHz for 5-10 minutes at 42°C (i.e., the transition temperature of the lipid) ^34^. We used the same method for preparing the PZQ-encapsulated niosomes by adding 500 mg of PZQ to Span 60 and cholesterol from the first step. 

For the lyophilization of the PZQ-encapsulated niosomes, we placed the PZQ-niosome solutions in 50 ml tubes, froze them in liquid nitrogen, and freeze-dried them using a vacuum freeze-drying machine at a pressure of 26.5 pascals. The lyophilized particles were then characterized ^35^.

### Characterization of PZQ-encapsulated niosomes

*Particle size analyzer.* We determined the distributing by size of the PZQ- encapsulated niosome particles using laser light scattering on a Beckman coulter particle size analyzer. PZQ-encapsulated niosomes were added to the sample dispersion unit containing the stirrer and stirred to reduce the aggregation between them while the laser obscuration range was maintained at 15-20%. The mean particle size was measured after performing the experiment in triplicate ^36^.

*Transmission electron microscopy (TEM).* The physical size and shape of the prepared PZQ-encapsulated niosomes were determined using a transmission electron microscope. For this purpose, the particle suspension was diluted 10 times with distilled water, deposited dropwise onto a 400-mesh copper grid coated with carbon film, and allowed to dry in the air before examining it under the microscope ^37^.

*Zeta potential.* The zeta potential (a key indicator of colloidal dispersion stability) of the PZQ-encapsulated niosomes dispersed in phosphate buffer solution (pH 6.5) was determined via laser Doppler anemometry. The nanovesicle suspension was diluted to 4 ml with a phosphate buffer (pH 6.5). An electric field of 150 millivolts (mV) was applied to observe the electrophoretic velocity of the vesicles. All measurements were made at 25 ºC in triplicate at the same ionic concentration ^35^.

### Determination of the encapsulation efficiency of niosomes

The encapsulation efficiency (EE) of PZQ was expressed as a ratio between the PZQ concentration in the niosomes and the concentration of PZQ added to the system. The amount of PZQ in the niosomes was determined by recording the absorbance of the loading solution at λ_max_= 490 nm (after removing niosomes by centrifugation at 10,000 rpm for 30 minutes). The unloaded PZQ in the supernatant was determined using a spectrophotometer at λ_max_ 490 nm. Niosomes EE was calculated according to the following equation: EE = [(D_t_-D_u_)/D_t_] x 100, where D_t_ represents the total amount of drug and D_u_ the amount of free drug ^38^.

### Experimental design

One hundred and sixty laboratory-bred Swiss strain albino mice aged 4-6 weeks weighing 20-30 g were individually infected with 100 cercariae using the paddling tail-immersion technique based on the method described by Smithers, *et al.*^39^. The mice were divided equally into two main groups: In Group I, mice were infected with *S. mansoni* susceptible cercariae from PZQ-unexposed snails (group a) and in Group II, mice were infected with *S. mansoni* cercariae with reduced sensitivity to PZQ from PZQ-exposed snails (group B). Each group was further subdivided equally into four main subgroups: Infected non-treated mice (subgroup a); infected PZQ-treated mice (subgroup b, where each animal was inoculated with a single oral dose of 500 mg/kg in two divided doses on the same day); infected niosomes-treated mice (subgroup c), and infected PZQ-niosomes-nanoparticles-treated mice (subgroup d). Mice treated with niosomes or PZQ-encapsulated niosomes were inoculated orally with a 400 µl single therapy of the prepared suspension divided into two doses on the same day.

Drugs were administrated against the adult *S. mansoni* stage 42 days after infection. All mice were perfused from the hepatic and mesenteric vessels 49 days after the cercarial challenge ^39^. The experiment was repeated thrice. We present the data from one of the independent experiments based on the average replicate.

We used the following parameters for assessing drugs against PZQ- susceptible and *S. mansoni* adult isolates with reduced sensitivity to PZQ:

To establish adults worm burden, we counted and sexed recovered worms under a stereo microscope.

For tissue egg count, we counted the eggs in both the liver and the intestine using Cheever›s technique ^40^. The mice liver and intestine were digested by overnight incubation in 4% (w/v) potassium hydroxide at 37°C. The digested tissue suspensions were thoroughly stirred and eggs were counted in 2x50 µl samples on microscope slides under 10x magnification. The mean of the duplicate egg count was determined for each mouse. The treatment-induced percentage reductions in worm and egg burdens were calculated as P = (C-V/C) x100, where P is the percentage of worm/egg counts reduction; C is the mean number of worms/eggs recovered from control mice, and V is the mean number of worms/eggs recovered from the treated mice.

The egg developmental stages (oogram pattern) were established to assess the therapeutic efficacy based on quantitative and qualitative oogram techniques following the criteria described by Pellegrino, *et al.*^41^. After perfusion of the portal system, 5-10 cm of the middle portion of the small intestine was opened longitudinally with a pair of scissors and rinsed in a petri dish of saline solution; three 10 mm fragments were cut off and processed for the oogram. We counted 100 eggs in each fragment and classified them according to the different stages of development as immature, mature, or dead.

### Statistical analysis

We used the statistical package for social sciences (SPSS) version 20.0 both for data presentation and statistical analysis of the results. Results were expressed as arithmetic mean, standard deviation (SD), minimum (Min.), maximum (Max.), and median; *p* equal to or less than 0.05 (p ≤ 0.05) was determined as the significance level. The Kolmogorov-Smirnov test was used to verify the normality of distribution. The Kruskal Wallis test was used for abnormally distributed quantitative variables in comparisons of more than two studied groups while the Mann Whitney test was used for pairwise comparisons ^42^.

## Results

### In vitro cercarial response to PZQ

**Figure 2:**
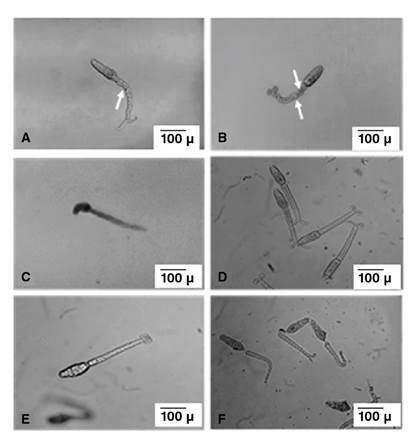
*In vitro* cercarial response to PZQ (x100) (scale bar 100μ). Cercariae harvested from group B snails showing; (A): tail constrictions (arrow) with two coiled rami of the bifurcation; (B): cercariae showing tail constrictions (arrows) with no coiling of the ramus of the bifurcation; (C): cercariae showed tilting of the head (stage 3); (D): cercariae showing disappearance of tail constrictions (stage 4) with two rami of the bifurcation coiled; (E): cercariae showing disappearance of tail constrictions (stage 4) with no coiling of ramus of the bifurcation; (F): dead cercariae showing separation of the tails (stage 5).

The onset of the first cercarial re-shedding from PZQ-exposed snails occurred 8 weeks after the cessation of drug exposure. The effect of PZQ on the harvested cercariae from the unexposed and exposed groups (group A and group B) was observed and classified into three stages: Stage 1 in which the unaffected live cercariae demonstrated normal swimming activity (rapid linear progressive swimming) and exhibited body contractions during movement with or without coiling of bifurcation rami (figure 2, A and B). In stage 2, affected live cercariae demonstrated intermittent spins (cercariae remained momentarily motionless before the body and tail suddenly rotated around their central axis with no linear progressive movement), tilted head (cercaria weakly moving their heads or tails without any progressive movement) (figure 2C), and sluggish head movement with complete absence of body movement. A loss of body contractions occurred with or without coiling of bifurcation rami (figure 2, D and E), and in stage 3, dead cercariae showed complete absence of head and tail movement. Some cercariae exhibited separation of the tail (figure 2F).

At the beginning of the experiment, the re-shed cercariae from PZQ- exposed snails in group B revealed no detectable differences regarding the cercarial activity compared with cercariae harvested from snails in group A (stage 1). Immediately after adding PZQ, an initial increase in the cercarial activity of snails in group B (prompt linear progressive and zigzag swimming motions) was observed for approximately 20 seconds. At the various examination points, statistically significant differences were recorded between cercariae harvested from both snail groups (group A and group B).

After the first 10 minutes, 91.67% of cercariae from the group B snails were completely unaffected (stage 1) with no recorded cercarial deaths while the remaining cercariae exhibited intermittent spins (stage 2). In contrast, after the same duration, 50.92% of cercariae from PZQ-unexposed snails (group A) died (stage 3). The cercarial death percentage in the same group increased up to 87.3% after 20 minutes of drug exposure with total cercarial deaths occurring at the 30-minute examination point. On the other hand, by increasing the duration of drug exposure, the influence of PZQ on the harvested cercariae from the PZQ-exposed group (group B) displayed slow and gradual enhancing effects.

**Figure 3:**
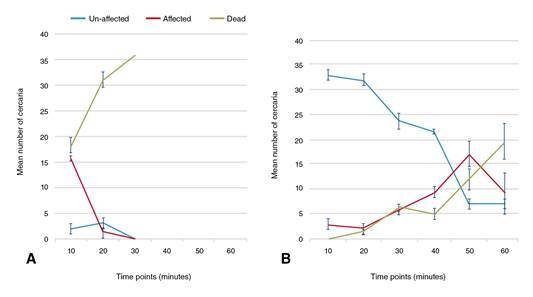
*In vitro* effect of 5 x 10-6 M PZQ on the cercariae (n=36) harvested from (A): PZQ unexposed control group (group A) and (B): PZQ exposed (group B) snails at a dose of 200 mg/kg PZQ twice/week followed by an interval of one week, and then repeated twice in the same manner. Lines represent mean count of un-affected, affected and dead cercariae after drug exposure every ten minutes for one hour. Error bars represent standard deviations of three cercarial sets per group.

After 30 minutes, up to 65.75% of the cercariae harvested from group B (PZQ-exposed snails) remained unaffected. The sum of the affected and dead cercariae markedly increased at the 50-minute and 60-minute examination points reaching approximately 80.55% at both points. At these examination points, 33.33% and 54.64% of the cercariae, respectively, were dead (figure 3).

### Characterization of PZQ-encapsulated niosomes

**Figure 4:**
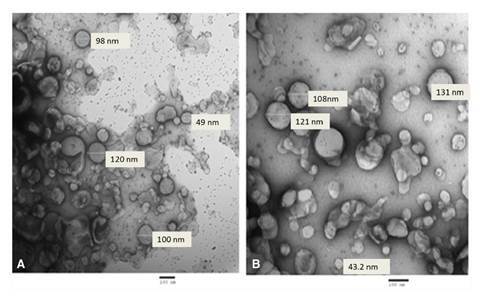
Transmission electron microscope of the nanoparticles: (A): rounded smooth surface of free niosomes with average size of 69.9-120 nm (5,000X); (B): PZQ encapsulated niosomes with average size of 43.2 -131 nm (5,000X).

The TEM of the prepared niosomes and PZQ-encapsulated niosomes revealed spherical, smooth surface nanoparticles with average sizes of 69.9-120 nm (figure 4A) and 43.2-131 nm (figure 4B), respectively**.** The zeta potential of the niosomes was -16.7 mV while that of the PZQ-encapsulated niosomes was -20.2 mV. Besides, the particle size distribution performed by the particle size analyzer revealed the niosomes without PZQ to have a mean particle size of 83.55 nm and showed a polydispersity index of 0.715 (i.e., less than 1) indicating the homogeneous nature of the formulation. On the other hand, the particle size distribution of the PZQ-encapsulated niosomes revealed a mean particle size of 97.14 nm and the polydispersity index was 0.534 (i.e., less than 1).

The PZQ encapsulation efficiency was expressed as the ratio of the PZQ concentration in the niosomes and the PZQ concentration added to the system. This formula produced a percentage of encapsulation efficiency of 66.8%.

### In vivo efficacy of PZQ and PZQ-encapsulated nanoparticles on adult S. mansoni

*Adults worm burden.* Recovered worms collected 49 days p.i. from all subgroups were counted and sexed under a dissecting microscope. A marked reduction of the drug potency was detected against adults collected from PZQ-treated mice infected with cercariae with reduced sensitivity to PZQ (subgroup IIb) compared with the control (non-treated mice infected with cercariae and reduced sensitivity to PZQ, subgroup IIa) and total worm reduction of 35.1%. This result was statistically non-significant (p>0.05) whereas under the same circumstances, the reduction of the female adult worm burden was statistically significant (47.37%; p≤0.05) (table 1).

**Table 1:** Total and female adult *S. manson*i worm loads among the different studied subgroups

Subgroups	Group I (susceptible group)				Group II (group with reduced sensitivity to PZQ)			
Worm Load	Ia (Control)	Ib	Ic	Id	IIa (Control)	IIb	IIc	IId
Total worm load								
Mean ± SD	30.83 ± 9.43	3.0 ± 1.55	30.0 ± 5.10	1.55 ± 0.94	22.5 ± 6.06	14.60 ± 3.30	21.2 ± 2.32	1.33 ± 0.52
Pcontrol		0.004*	0.747	<0.001*		0.009*	0.259	0.003*
Sig. bet. Groups						p_4_=0.001*, p_5_<0.001*, p_6_=0.003**		
Sig. bet. Groups	p_7_= 0.092, p_8_<0.001*, p_9_= 0.004*, p_10_=0.645							
% Reduction		↓90.27	↓2.69	↓95.0		↓35.1	↓5.78	↓94.09
Female worm load								
Mean ± SD	15.7 ± 4.4	1.5 ± 0.84	14.0 ± 2.68	1.10 ± 0.79	13.3 ± 2.2	7.0 ± 5.6	12.2 ± 3.5	1.33 ± 0.52
Pcontrol		0.003*	0.744	<0.001*		0.024*	0.220	0.003*
Sig. bet. Groups		p_4_= 0.053, p_5_= 0.182, p_6_= 0.003*				p_4_= 0.053, p_5_= 0.182, p_6_= 0.003*		
Sig. bet. Groups	p_7_= 0.255, p_8_= 0.190, p_9_= 0.170, p_10_=0.553							
% Reduction		↓90.45	↓10.83	↓93.0		↓47.37	↓8.27	↓90.0

Group I (susceptible group): mice infected with PZQ susceptible cercariaee; Ia: non treated; Ib: PZQ treated; Ic: niosomes nanoparticles treated; Id: PZQ niosomes nanoparticles treated.

Group II (group with reduced sensitivity to PZQ): mice infected with cercariae with reduced sensitivity to PZQ; IIa: non treated; IIb: PZQ treated; IIc: niosomes nanoparticles treated; IId: PZQ niosomes nanoparticles treated.

% Reduction: Percentage reduction between each subgroup and the control subgroup

P: Kruskal Wallis test, Significance between groups was done using Mann Whitney test.

Pcontrol: p value for comparing between Control and each subgroup

p1: p value for comparing between Ib and Ic

p2: p value for comparing between Ib and Id

p3: p value for comparing between Ic and Id

p4: p value for comparing between IIb and IIc

p5: p value for comparing between IIb and IId

p6: p value for comparing between IIc and IId

p7: p value for comparing between Ia and IIa

p8: p value for comparing between Ib and IIb

p9: p value for comparing between Ic and IIc

p10: p value for comparing between Id and IId

No activity of niosome nanoparticles against the adult stage was detected in niosomes-treated mice infected with cercariae either PZQ-susceptible or with reduced sensitivity to PZQ (subgroups Ic and IIc) compared with their controls (subgroups Ia and IIa). The percentage reductions were 2.69% and 5.78%, respectively (statistically non-significant: p>0.05) while oral treatments with PZQ-encapsulated niosomes (subgroups Id and IId) revealed the highest drug efficacy among all the studied subgroups. Highly significant reductions were obtained by comparing subgroups Id and IId with their controls (subgroups Ia and IIa). The mean total worm load was 1.33 ± 0.52 with a statistically significant reduction of 94.09% and the complete elimination of adult male worms was detected in *S. mansoni* with reduced sensitivity to PZQ treated with PZQ-encapsulated niosomes (subgroup IId) (p≤0.05). However, in subgroup Id (PZQ-susceptible *S. mansoni* treated with PZQ-encapsulated niosomes), a slight enhancement of the drug efficacy was reported with a statistically significant reduction of 95% (p ≤ 0.05).

### Tissue egg count and oogram patterns

Niosomes-treated mice infected with PZQ-susceptible cercariae or cercariae with reduced sensitivity to PZQ (subgroups Ic and IIc, respectively), did not reveal statistically significant differences in each total egg count in liver and intestinal tissues and their oogram patterns (*p*˃0.05). However, oral treatment with PZQ or PZQ-encapsulated niosomes in either the mice infected with PZQ-susceptible cercariae (subgroups Ib and Id) or the mice infected with cercariae with reduced sensitivity to PZQ (subgroups IIb and IId) demonstrated statistically significant reductions in the egg counts in both hepatic and intestinal tissues compared with those of their control subgroups (p≤0.05) (table 2).

**Table 2:** Hepatic and intestinal tissue egg counts (x10^2^) per gram of tissue among the different studied subgroups

Subgroups	Group I (susceptible group)				Group II (group with reduced sensitivity to PZQ)			
Egg counts	Ia (Control)	Ib	Ic	Id	IIa (Control)	IIb	IIc	IId
Liver egg count								
Mean ± SD	347.2±157.98	24.67 ± 32.05	244.2 ± 55.4	18.79 ± 1.81	193.8 ± 60.8	67.8 ± 33.4	139.2 ± 117.7	4.50 ± 3.6
Pcontrol		0.004*	0.150	<0.001*		0.008*	0.109	0.004*
Sig. bet. Groups		p_1_=0.004*, p_2_=0.223, p_3_<0.001*				p_4_=0.078, p_5_=0.004*, p_6_=0.004*		
Sig. bet. Groups			p_7_= 0.016*, p_8_= 0.025*, p_9_= 0.078, p_10_<0.001*					
% Reduction		↓89.54	↓29.67	↓94.59		↓65.02	↓28.17	↓97.68
Intestinal egg count								
Mean ± SD	384.7 ± 136.7	57.3 ± 32.9	242.3 ± 102.7	42.30 ± 5.47	231.67 ± 74.4	137.7 ± 38.02	222.7 ± 73.1	3.33 ± 1.8
Pcontrol		0.004*	0.055	<0.001*		0.037*	0.687	0.004*
Sig. bet. Groups		p_1_=0.004*, p_2_=0.031*, p_3_<0.001*				p_4_=0.055, p_5_=0.004*, p_6_=0.004*		
Sig. bet. Groups			p_7_= 0.037*, p_8_= 0.010*, p_9_= 1.000, p_10_<0.001*					
% Reduction		↓85.12	↓37.02	↓89.0		↓40.56	↓3.87	↓98.56

Group I (susceptible group): mice infected with PZQ susceptible cercariaee; Ia: non treated; Ib: PZQ treated; Ic: niosomes nanoparticles treated; Id: PZQ

niosomes nanoparticles treated.

Group with reduced sensitivity to PZQ (group II): mice infected with cercariae with reduced sensitivity to PZQ; IIa: non treated; IIb: PZQ treated; IIc: niosomes

nanoparticles treat ed; **IId:** PZQ niosomes nanoparticles treated.

% Reduction: Percentage reduction between each subgroup and the control subgroup

P: Kruskal Wallis test, Significance between groups was done using Mann Whitney test.

Pcontrol: p value for comparing between Control and each subgroup

p1: p value for comparing between Ib and Ic

p2: p value for comparing between Ib and Id

p3: p value for comparing between Ic and Id

p4: p value for comparing between IIb and IIc

p5: p value for comparing between IIb and IId

p6: p value for comparing between IIc and IId

p7: p value for comparing between Ia and IIa

p8: p value for comparing between Ib and IIb

p9: p value for comparing between Ic and IIc

p10: p value for comparing between Id and IId

* Statistically significant at P ≤ 0.05

For the PZQ-susceptible isolate, PZQ and PZQ-encapsulated niosomes- treated mice (subgroups Ib and Id) revealed a statistically significant increase in the dead eggs with mean percentages of 47.67% and 69.90%, respectively, and a statistically significant reduction in the immature eggs with mean percentages of 11.83% and 8.95%, respectively (p≤0.05) (table 3).

**Table 3:** Oogram pattern (Egg developmental stages) of the different studied subgroups

Subgroups	Group I (susceptible group)				Group II (group with reduced sensitivity to PZQ)			
Egg counts	Ia (Control)	Ib	Ic	Id	IIa (Control)	IIb	IIc	IId
Liver egg count								
Mean ± SD	33.92 ± 4.45	40.0 ± 20.12	27.50 ± 9.29	21.15 ± 5.93	35.33 ± 29.24	32.33 ± 16.26	45.67 ± 21.51	12.33 ± 11.71
Pcontrol		0.054	0.261	<0.001*		0.748	0.337	0.024*
Sig. bet. Groups		p_1_=0.054, p_2_=0.014*, p_3_<0.001*				p_4_=0.748, p_5_=0.337, p_6_=0.423		
Sig. bet. Groups			p_7_=0.337, p_8_=0.520, p_9_=0.128, p_10_=0.027*					
Immature eggs								
Mean ± SD	63.77 ± 4.94	11.83 ± 9.62	66.17 ± 6.11	8.95 ± 3.33	59.17 ± 26.57	29.50 ± 26.24	51.0 ± 22.12	0.0 ± 0.0
Pcontrol		0.004*	0.469	<0.001*		0.045*	0.520	0.002*
Sig. bet. Groups		p_1_=0.004*, p_2_=0.575, p_3_<0.001*				p_4_=0.045*, p_5_=0.520, p_6_=0.199		
Sig. bet. Groups			p_7_=0.520, p_8_=0.200, p_9_=0.335, p_10_<0.001*					
Dead eggs								
Mean ± SD	2.32 ± 1.80	47.67 ± 28.52	6.67 ± 5.47	69.90 ± 5.19	5.67 ± 3.56	32.17 ± 22.52	3.33 ± 1.51	87.67 ± 16.93
Pcontrol		0.004*	0.090	<0.001*		0.043*	0.168	0.004*
Sig. bet. Groups		p_1_=0.006*, p_2_=0.014*, p_3_<0.001*				p_4_=0.036*, p_5_=0.168, p_6_=0.054		
Sig. bet. Groups			p_7_=0.049*, p_8_=0.423, p_9_=0.257, p_10_=0.123					

Group I (susceptible group): mice infected with PZQ susceptible cercariae; Ia: non treated; Ib: PZQ treated; Ic: niosomes nanoparticles treated; Id: PZQ niosomes

nanoparticles treated.

Group II (Group with reduced sensitivity to PZQ): mice infected with cercariae with reduced sensitivity to PZQ; IIa: non treated; IIb: PZQ treated; IIc: niosomes

nanoparticles treated; IId: PZQ niosomes nanoparticles treated.

% Reduction: Percentage reduction between each subgroup and the control subgroup

P: Kruskal Wallis test, Significance between groups was done using Mann Whitney test.

Pcontrol: p value for comparing between Control and each subgroup

p1: p value for comparing between Ib and Ic

p2: p value for comparing between Ib and Id

p3: p value for comparing between Ic and Id

p4: p value for comparing between IIb and IIc

p5: p value for comparing between IIb and IId

p6: p value for comparing between IIc and IId

p7: p value for comparing between Ia and IIa

p8: p value for comparing between Ib and IIb

p9: p value for comparing between Ic and IIc

p10: p value for comparing between Id and IId

*: Statistically significant at P ≤ 0.05

On the other hand, mice infected with less PZQ-sensitive cercariae orally treated with PZQ (subgroup IIb) exhibited a much lower reduction in the number of immature eggs or an increase in the number of dead eggs. No significant changes in the percentage of the mature eggs were detected in subgroup IIb compared with subgroup IIa (p>0.05). However, a remarkable increase in the percentage of dead eggs was detected in subgroup IId animals receiving PZQ-encapsulated niosomes with a mean percentage of 87.67% ± 16.93.

## Discussion

In the present work, we obtained schistosomes with reduced sensitivity to PZQ by drug selection during the asexual stages of the parasite in the snails. Shedding snails were treated with PZQ doses of 200 mg/kg twice/ week followed by an interval of one week, then repeated twice in the same manner, and reared in glass containers changing the DTW every 24 hours. This approach is far less expensive, time-consuming, and labor-intensive than other strategies applying drug pressure through multiple intra-mammalian stage passages ^10^. This regimen and rearing conditions decreased the stress to which the snails were exposed by dividing the dose of the drug and decreasing the time for the protein content of the paste in water to be fermented, especially in a warm climate. Additionally, glass containers might offer a suitable temperature for snail growth and survival. Glass, for instance, is a very good insulator at room temperature but becomes a conductor only when heated at very high temperatures. It is worth mentioning that the approximate temperature for snail breeding and reproduction is 15 to 25 °C; snails cannot survive at > 29 °C and may die within several hours at > 40 °C ^43^.

In PZQ-exposed snails, cercarial shedding ceased for eight weeks after the termination of drug exposure. According to Mattos, *et al.*^44^, this may be partially explained by the use of a sublethal dose of PZQ which in their study induced morphological and metabolic alterations on the sporocysts, thereby interrupting the shedding. As the effect of PZQ on the sporocysts is temporary and reversible ^33^, re-shedding of the cercariae occurred after recovery.

The successful inducing of reduced sensitivity to PZQ in the present study was confirmed *in vitro* by reducing the PZQ susceptibility of cercariae harvested from the PZQ-exposed snails compared to those from the PZQ- unexposed snails. Similarly, cercariae from snails infected by resistant isolates induced either experimentally or collected from the field exhibited reduced susceptibility to PZQ ^32^^,^^45^. Furthermore, the development of reduced sensitivity to PZQ was confirmed experimentally. The therapeutic efficacy of 500 mg/kg PZQ was greatly diminished and the 35.1% reduction in the total worm load in treated mice infected with cercariae with reduced sensitivity to PZQ (subgroup IIb) was not significant compared with its control. According to Coles, *et al.*^46^, this value for worm recovery is sufficient to provide an isolate resistant to PZQ.

Parasite and/or host factors could serve as underlying reasons for such unresponsiveness. In schistosomes, PZQ tolerance has been linked to over-expression of sarco/endoplasmic reticulum Ca ^2+^ ATPases, heat shock protein 70 in addition to glycoproteins, or other multidrug transporters leading to increased drug efflux ^13^^,^^47^^,^^48^. In their study on the possible host factors involved in the PZQ unresponsiveness, Hanallah, *et al.*^49^ reported that PZQ insusceptible *S. mansoni* isolates possess a different immunogenic makeup both qualitatively and/or quantitatively when compared to isolates susceptible to PZQ. Furthermore, according to Botros, *et al*. ^50^, the decreased sensitivity to PZQ could be due to a lower inhibition of hepatic drug-metabolizing enzymes, mainly cytochrome P 450 (CYP 450) in hosts infected with resistant *Schistosoma* isolates with a consequently higher metabolic transformation of PZQ and lower level of serum drug concentration. The inhibition of the activity of hepatic drugs metabolizing enzymes in PZQ susceptible *S. mansoni-*infected mice was previously reported ^51^. Such inhibition was attributed to the possible denaturation of CYP 450 to its inactive form (CYP 422) as a result of the inflammatory reaction following egg deposition ^52^. Therefore, lower inhibition of such enzyme could occur in association with the demonstrated significant reduction of the hepatic egg counts in the *S. mansoni* resistant isolate.

When mice infected with *S. mansoni* isolates with reduced sensitivity to PZQ were treated with PZQ, a significant reduction in the female worm load in subgroup IIb compared with its control was demonstrated. The sex-specific sensitivity between male and female schistosomes could be explained by the fact that these are more metabolically active than those of males. Interestingly, Kasinathan, *et al*. ^12^ revealed an expression of higher levels of *S. mansoni* multidrug resistance-like protein transporters in males than in female worms responsible for resistance. This can consequently explain the significant egg count reduction in the livers and intestines of mice infected with *S. mansoni* isolates having reduced sensitivity to PZQ and treated with PZQ (subgroup IIb) despite the non-significant reduction in the total worm load compared with their control (subgroup IIa).

We reported the lack of any significant therapeutic effect of niosomes nanoparticles (NPs) orally administrated alone against adult *S. mansoni* with reduced sensitivity to PZQ. In contrast to metals, metal oxides, and polymer- based NPs (well known for their highly potent antimicrobial effect), biological nanoparticles such as lipids are used only for drug delivery ^13^^,^^18^^,^^53^.

Regarding niosomes safety, it is generally believed that lipids are biocompatible. A niosome is a non-ionic surfactant-based liposome. They are formed mainly by cholesterol incorporation as an excipient. In our study, Span 60 was the non-ionic surfactant used for preparing the niosome particles. Span 60 is one of the alkyl esters, which are considered non-toxic and non-irritant materials ^54^. The Food and Drug Administration revised them in 2015 as one of the food additives ^55^. These biophysical properties of niosomes support their tissue non-toxicity.

Oral administration of niosomes in conjugation with PZQ against susceptible adult *S. mansoni* (subgroup Id) enhanced the therapeutic efficacy of the drug as to the studied parasitological parameters. Due to the presence of hydrophilic, amphiphilic, and lipophilic moieties in their structure, drug molecules with a wide range of solubility can be accommodated. These moieties may act as a depot, releasing the drug in a controlled manner. Similarly, solid lipid nanoparticles loaded with PZQ were effective in reducing the worm load and the tissue egg count ^56^. In our study, niosomes were able to promote a high concentration of the drugs in the target cells and when combined with sodium stibogluconate they were found to be more effective than liposomes against experimental murine visceral leishmaniasis ^57^.

The low aqueous solubility of PZQ is considered a limiting factor as regards its bioavailability ^58^. On the other hand, niosomal drug delivery enhances drug bioavailability by crossing the anatomical barrier of the gastrointestinal tract via cell transcytosis of Peyer’s patches in the intestinal lymphatic tissues ^23^.

Here, we demonstrated that the regimen using single therapy of PZQ- encapsulated niosome in mice infected with cercariae with reduced sensitivity to PZQ was capable of successfully overcoming the tolerance of *S. mansoni* to PZQ as revealed by our results displaying statistically significant reductions in all the evaluated indicators of drug susceptibility compared with all the relevant studied subgroups. Kulsirirat, *et al.*^26^ demonstrated that non-ionic surfactants had an inhibitory effect on P-gp ATPase activity transporters, which have been linked to PZQ resistance in schistosomes ^12^. This finding identified a possible mechanism by which niosomes could overcome the reduced sensitivity to PZQ. In agreement with the reported data, the development of new therapeutics or compounds targeting these transporters proved useful in enhancing the efficacy of the drugs ^6^^,^^59^. Verapamil and tariquidar are considered P-gp inhibitors and can enhance the cytotoxic effects of chemotherapeutic drugs against resistant parasites ^6^^,^^60^. Moreover, a combination of the P-gp transporter inhibitors and PZQ has been successfully used against resistant *S. mansoni*^9^^,^^59^.

We report here how PZQ-encapsulated niosomes enhanced and restored drug sensitivity against susceptible and laboratory-induced *S. mansoni* adult worms with reduced sensitivity to PZQ, respectively. Interestingly, the primary mechanism of overcoming the drug resistance could be related to the surfactant inhibitory effect on P-gp efflux transporters. Further investigations are necessary to verify the exact mechanisms by which niosomal nanoparticles exert their effect against tolerant parasites.
